# A Review of the Effect of COVID-19-Related Lockdowns on Global Cancer Screening

**DOI:** 10.7759/cureus.40268

**Published:** 2023-06-11

**Authors:** Annika Bharwani, Dan Li, Sten H Vermund

**Affiliations:** 1 Public Health, Yale University, New Haven, USA

**Keywords:** covid 19 impact of lockdown, covid-19 lockdown, public health policies, covid-19 and cancer screening, covid 19, global oncology, public health care, preventive oncology

## Abstract

COVID-19 lockdowns have led to significant disruptions in preventative health services worldwide. This review aims to assess the impact of COVID-19 lockdowns on worldwide preventive cancer screening participation. Major medical databases were searched using the keywords ‘lockdown,’ ‘cancer,’ and ‘screening or diagnosis,’ and relevant articles were evaluated against inclusion and exclusion criteria.* *The final review consisted of 38 studies. The impact of COVID-19 on screening uptake was categorized based on cancer type. All types of screening had decreased participation during or around the lockdown period. Racial and socioeconomic disparities, provider-related barriers, and patient attitudes about service disruptions during the pandemic were also highlighted in this review. Future research should focus on data from low- and middle-income countries to obtain a more comprehensive picture of the problem. Policy interventions that adopt self-screening or different screening intervals can also be considered to reduce impacts in future crises. Insights from existing studies and future research will allow for more proactive measures to manage future disruptions.

## Introduction and background

Introduction

Cancer is one of the most prevalent non-communicable diseases and a leading cause of death globally [1]. The rate of new cancer cases has increased steadily between 2010 and 2019, leading to an estimated 250 million disability-adjusted life years in 2019, 96.9% of which arise from years of life lost [2]. While some cancers contribute to higher mortality than others, most common cancers have much better outcomes when detected early enough that the disease is still localized [3].

When utilized appropriately, screening can save lives by detecting early-stage tumors. A recent study analyzing patients in Canada and Europe demonstrated that women exposed to breast cancer screening had a 40% lower mortality risk [4]. Furthermore, screening for multiple types of cancers in asymptomatic, high-risk individuals is also cost-effective and possibly even cost-saving [5]. Many countries have already established cancer screening protocols for common cancers to help reduce the burden of late-stage disease. The United States, for instance, has national screening recommendations for breast, cervical, colorectal, and lung cancers in place [6].

During the COVID-19 pandemic, many countries went into lockdown to reduce case transmission, minimize patient fatalities, and reduce overstretching of the medical system [7,8]. Aside from closing down social gathering places, schools, and workplaces, many nonessential preventative healthcare services were suspended or reduced significantly so that healthcare staff could care for COVID-19 patients. Despite the importance of these measures in controlling the pandemic, there are associated health consequences, such as a reduction in cancer screening services.

The primary outcome of this review is to assess the impact of COVID-19-related lockdowns on global cancer screening. The secondary outcomes of this study are to explore disparities in screening disruption, changes in the number of screen-detected tumors (i.e., tumors that were detected solely through screening and were otherwise asymptomatic), provision-related barriers to screening (i.e., barriers in staffing, equipment supply, clinic operation, etc.), and patient attitudes toward screening.

Methods

Search Strategy

A literature search was conducted using three major medical databases: Scopus, Ovid, and PubMed looking at articles published between January 1, 2020 and November 6, 2022. An initial search was conducted on PubMed using the MeSH terms ‘cancer,’ ‘lockdown*,’ ‘screening,' or diagnosis,’ and all articles had to include ‘screening’ in the title. Articles from PubMed were run through Research Rabbit, which retrieved other related articles. For comprehensiveness, two further searches were conducted in Ovid and Scopus with MeSH terms ‘cancer,’ ‘lockdown,’ ‘screening or diagnosis,’ and had to include ‘screening’ in the article title. Only English articles were considered for primary review. 

Inclusion and Exclusion Criteria

Inclusion criteria were that articles had to come from peer-reviewed journals, specifically discussing the effect of COVID-19 lockdowns on cancer screening, and consist of actual quantitative or qualitative data. As a result, the study types included were reviews, meta-analyses, cohort studies, cross-sectional studies, qualitative studies, and mixed-methods studies. Exclusion criteria included articles without full text, those that measured outcomes through invasive diagnostic procedures or utilized modeling data, studies that were commentaries or editorials, and studies that consisted of data from regions that did not experience lockdown. For the purposes of this article, lockdown referred to countries that had any cessation of recreational or nonessential services and activities or a stay-at-home order during the COVID-19 pandemic. Online searches of COVID-19 world data banks and country-specific reporting determined region-specific inclusion. Articles from countries or regions that allowed limited activities in small groups were not included.

Article Selection Process

Retrieved abstracts were screened against the inclusion and exclusion criteria. Articles that specifically assessed the impact of COVID-19 lockdowns on cancer screening were considered relevant (e.g., patient satisfaction, patient wishes, the actual decrease in the number of screenings, and others). After the initial screening and the removal of duplicates, full texts were reviewed to ensure that included studies adhered to the inclusion and exclusion criteria.

Data Extraction

All full articles that adhered to the inclusion and exclusion criteria were reviewed again to assess critical details from the study. Articles were reviewed to assess the countries from which the data was collected, the method of study, objectives, inclusion and exclusion criteria if applicable, primary and secondary outcome measures, types of screenings, and study outcomes.

## Review

Results

Description of Studies Included for Review

**Figure 1:**
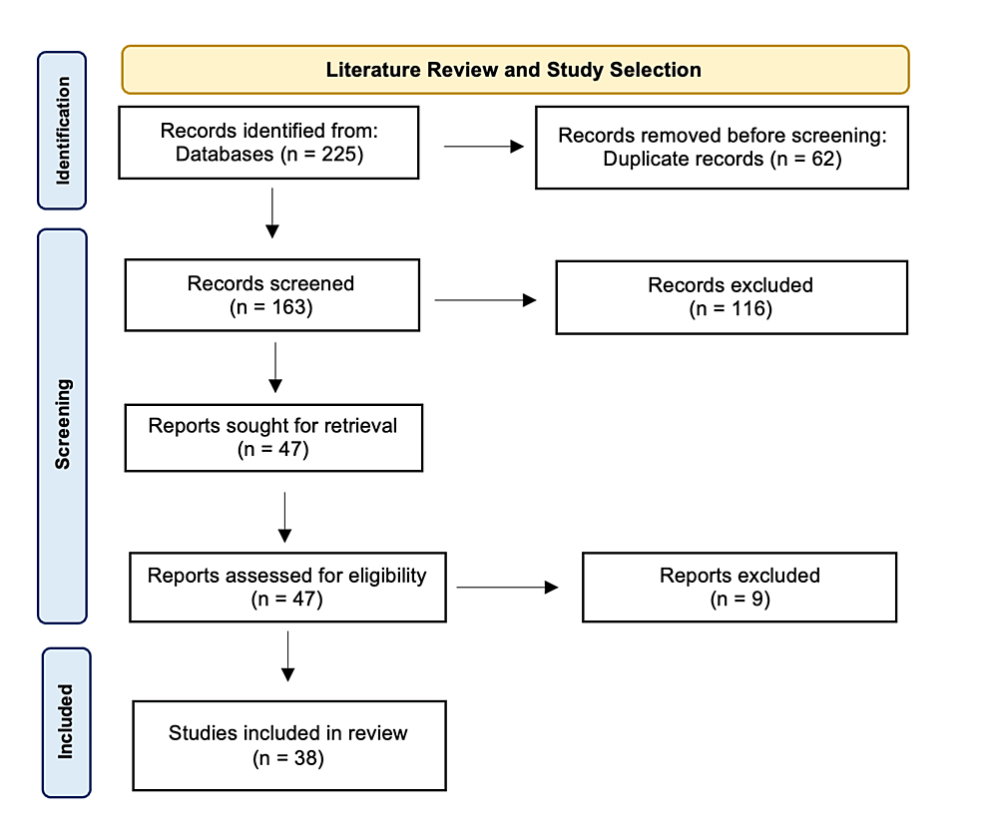
PRISMA flowchart for study selection PRISMA: Preferred Reporting Items for Systematic Reviews and Meta-Analyses

The initial literature search yielded 225 individual articles (PubMed = 21, Scopus = 31, Ovid = 65, Research Rabbit = 108). After duplicates were removed (n = 62), 163 titles and abstracts were reviewed against the inclusion and exclusion criteria. After a preliminary review, 47 relevant articles were identified for a full-text review. Nine studies were excluded after the final review as they did not meet the inclusion criteria (e.g., not focused on screening, data from a country that did not experience lockdown, not yet peer-reviewed, or no full text available). The final review included 38 articles (Figure 1).

Study Designs

Of the 38 papers included in this analysis, 11 were reviews [9-19], 22 were cohort studies [20-41], four were cross-sectional studies [42-45], and one was a mixed-methods study [46]. The reviews consisted of narrative reviews, short reviews, systematic reviews, and meta-analyses. Reviews looked at the impact of COVID-19 on changes in cancer screening uptake before and during the pandemic by drawing comparisons between multiple countries, multiple cancer types, or both. Cohort studies compared screening uptake during COVID-19 to the periods before and after the pandemic lockdown. These periods ranged from a few months before the onset of the pandemic to five years before the pandemic began. Some cross-sectional studies had data on the quantitative screening impact of the lockdown period, while others focused qualitatively on patient attitudes and provision-related barriers. The mixed-methods study consisted of a cross-sectional survey about patient attitudes coupled with more thorough interviews in a subsample of survey participants.

Countries Included and Demographics

The papers included were from multiple countries, with 13 from the United States [19,21,23-25,27-31,35,39,42], six from the United Kingdom [10,13,32,36,43,46], two from Italy [22,33] and the Netherlands [40,41], and one from Argentina [37], Bangladesh [17], Canada [34], Slovenia [38], Turkey [26], and Belgium [20], respectively. Some review articles assessed multiple countries that were not evaluated in individual papers. Most countries had their first cases of COVID-19 at some point in January and went into lockdown between mid-March to early April 2020 [7]. Of note, Italy experienced lockdowns in early March (March 9) 2020 [7], and Turkey went into lockdown on April 29, 2020 [8].

Screening Types and Characteristics

Fifteen of the studies looked at multiple types of cancer screening [9,11,15-17,19,20,22,23,28,34,36,37,39,44], eight looked specifically at breast cancer screening [12,21,24,26,27,35,40,41], seven looked at cervical cancer screening [10,13,18,29,38,43,45], three looked at colorectal cancer screening [14,30,32], and two looked at lung cancer screening [31,42]. One paper looked at breast and cervical cancer screening [25], one looked at cervical and colorectal cancer screening [46], and one looked at prostate cancer screening [33].

Screening Participation

Screening participation was the primary or secondary outcome in 32 of the studies. Studies consistently demonstrated significant decreases in screening rates corresponding to the start of the lockdown periods (Table 1). Overall, screening disruptions were more severe in countries that had poorly controlled COVID-19 outbreaks [9].

**Table 1:** Studies demonstrating the impact of COVID-19 lockdowns on screening uptake HPV: human papillomavirus, FOBT: fecal occult blood test, PSA: prostate-specific antigen, FIT: fecal immunochemical test, LMICs: low- and middle-income countries

Author	Country (region)	Approx. beginning of the lockdown period	Study design	Cancer type (screening type)	Main outcomes
Alkatout et al. [9]	Multiple countries	Various dates across different countries	Systematic review	Multiple types of cancer	The pandemic led to worldwide disruptions in cancer screenings, but the interruption was more severe in countries with poorly controlled COVID-19 infections. Colonoscopy rates fell by about 4.1%, while gastroscopies, prostate and lung, and mammogram screenings were reduced by 57%, 74%, 56%, and 22.2-85%, respectively. Cancer screening rates decreased at all care levels and in almost all age groups.
Masson [10]	United Kingdom (Scotland)	March 23, 2020	Review	Cervical cancer (pap smears)	Pre-pandemic, screening uptake was 71.2% of the eligible population, with a 10.5% difference in uptake between the most and least deprived areas of Scotland. Between November 1st, 2019, and October 31st, 2020, only 43% of the previous year’s screening tests were carried out. Overall, general practices across the region had a 56% in the number of tests carried out compared to previous years.
Teglia et al. [11]	Multiple countries	Various dates across different countries	Systematic review and meta-analysis	Breast, cervical, and colorectal cancer	Breast: The maximum decrease in breast cancer screening was a 74.3% decrease in April 2020. In contrast, there was no significant reduction in screening after June 2020. North America (Massachusetts United States and Canada) had a 44.6% decrease in breast cancer screening during the COVID period, with a maximum decrease of 86.7% in April. European studies demonstrated a more significant decrease in breast cancer screening than in North America. Colorectal: Overall screening of colorectal cancer went down by 44.9% compared to the pre-pandemic period, with a 51.5% decrease in clinic-based settings and a 43.7% decrease in population-based settings. The most significant decrease was in April (69.3%), with only partial improvement from June to October. Cervical: There was a significant decrease in cervical cancer screening in March compared to earlier periods (-78.8%). Clinics had a higher rate of reduction than population-based settings.
Ng and Hamilton [12]	18 different countries	Various dates across different countries	Rapid review and meta-analysis	Breast cancer (mammography)	Across countries, there was a 9.8% to 80.0% reduction in mammography screening volume. On average, mammography screening was reduced by about 41-53% between 2019 and 2020. There was a higher rate of reduction in countries with lockdown than those that did not have a complete lockdown (RR: 0.47 vs. 0.67 p = 0.03).
Leeson et al. [13].	United Kingdom (Wales)	March 23, 2020	Review	Cervical cancer (HPV-based primary screening)	All invitations were stopped between March 22 to June 22, 2020, with a few general practices continuing to take screening appointments. The temporary cessation of screening services involved about 148,000 women across Wales, with the main delay in diagnosis occurring in May and June for follow-up referrals.
Kopel et al. [14]	Multiple countries	Various dates across different countries	Systematic review	Colorectal cancer (flexible sigmoidoscopy and colonoscopy or stool-based tests)	LMICs such as Paraguay, Thailand, and Iran continued to offer colorectal screening tests, but they only operated at 20-90% of the volume expected pre-pandemic. Across the United Kingdom, there were low colorectal cancer screening rates throughout the pandemic. In the United States, colorectal screening rates were reduced by 79.3% in April 2020 compared to April the year before, with some resumption of service in June and July; however, it did not reach pre-pandemic levels. The highest reductions were seen in the Northeast regions among higher socioeconomic levels. Of note, the pandemic led to an interruption in developing screening in the colorectal cancer “hotspot” region in Appalachian Kentucky, leading to a significant backlog.
Osei et al. [15]	Multiple countries	Various dates across different countries	Narrative review	Multiple types of cancer	In the United States, weekly screenings for breast, cervical, and colorectal cancers decreased between 86% and 94% from January 20 to April 21, 2020.
Elkrief et al. [16]	Multiple countries	Various dates across different countries	Review	Multiple types of cancer	In the United Kingdom, all types of cancer screening decreased during the pandemic, with breast cancer screening having the most significant decrease (-90%), followed by colorectal cancer screening (-85%). In Canada and Scotland, researchers reported negative impacts on all cancer screening programs, with low neighborhood income and older age associated with diagnostic delays.
Basu et al. [17]	Bangladesh	March 26, 2020	Review	Multiple types of cancer	Pre-pandemic, many LMICs had suboptimal screening services. About 25% of LICs reported less than 10% coverage for breast cancer screening, and about 50% of LICs reported less than 10% coverage for cervical cancer screening. Many LMICs that claimed to have screening did not have proper plans for screening implementation. Bangladesh’s screening and diagnostic services were interrupted from March 23 to May 30, 2020. About 14.1% fewer women were screened for cancer in 2020 compared to 2018 and 2019. The lowest cancer screening rate among women was seen during April 2020, making up only 5.1% of women screened during the same period in the previous year. There was a gradual improvement in the screening volume in July 2020, but the country saw another downward trend in women screened after the second wave hit in 2021.
Sasidharanpillai and Ravishankar [18]	Multiple countries	Various dates across different countries	Systematic review and meta-analysis	Cervical cancer	During the pandemic, 4.24% of women were screened for cervical cancer compared to 9.79% in the pre-pandemic period.
Barsouk et al. [19]	United States	Late March 2020	Review	Multiple types of cancer	Across many developed nations, the rates of recommended cancer screening went down during the pandemic. By April 2020, across health networks in the United States, mammograms decreased by 89.2%. Cervical cancer screening rates through HPV testing and pap smears decreased by 87% (screening rates improved to a 40% decrease from baseline by June 2020), colorectal cancer screening decreased between 79% and 84.5%, and low-dose CTs went down by 72-78%.
Jidkova et al. [20]	Belgium	March 18, 2020	Cohort study	Breast, cervical, and colorectal cancer	Breast: Breast cancer screening programs were halted between March 23 and June 28, 2020. Breast cancer invitation coverage in 2020 (88.1%) was much lower than it was in 2019 (97.5%), with a backlog of 44,434 women receiving invitations by the end of 2020. Over half of those whose screenings were delayed received invitations in the first three months of 2021. Cervical: Cervical cancer screening services were halted between March 22 and May 23, 2020. Vertical screening invitations, however, were stable when averaging over the entire year of 2020 compared to 2019 despite some fluctuations throughout the year. Colorectal: Colorectal cancer screening services were halted between March 22 and May 23, 2020, and again between November 15 to November 28, 2020. During the lockdown, there was a drop in the number of people screened. After the first lockdown, there was a higher uptake of services. Overall, all age groups had minor decreases in colorectal screening after the first lockdown, except the 70-74 age group, which had a 20% increase in screening after the first lockdown.
Yin et al. [21]	United States	Late March 2020	Cohort study	Breast cancer (mammography, surgery, and genetic risk clinic)	Mammography began to decrease on March 15, with an initial drop of 51.3% compared to the week prior. On average, the weekly decline was 61.7%, with a maximal decline of 72.1% from March 15 to March 22, 2020. Screening reached a nadir on April 5, 2020, when only 5.4% of the pre-pandemic screening volume was screened.
Battisti et al. [22]	Italy	March 9, 2020	Cohort study	Breast (mammography), cervical (pap smear or HPV DNA), colorectal cancer (FOBT or sigmoidoscopy)	Throughout 2020 there were, on average, 26.6% fewer mammography invitations (range: -60% to -0.5%), 31.8% fewer colorectal cancer screening invitations (range: -70.5% to +6.8%), and 33% fewer cervical cancer screening invitations (range: -71.3% to +19.8%). Despite efforts by screening centers to recover delays, the recovery has been incomplete, and there is significant variation among screening centers. There has been a 15% decrease in mammography participation, a 15% decrease in cervical cancer screening participation, and a 20% decrease in colorectal cancer screening participation.
Bakouny et al. [23]	United States (Massachusetts)	March 23, 2020	Cohort study	Breast (mammography), Cervical (pap smear), colorectal (colonoscopy), lung (low-dose CT), prostate cancer (PSA)	During the three-month disruption, only 15,453 patients underwent any of the five cancer screenings offered, compared to 64,269 patients in the three months prior to the pandemic onset, and 51,944 patients in the three months after the initial pandemic period. Decreases in screening were accompanied by decreases in ensuring diagnosis across all five screening tests. For all screening tests except low-dose CT, there was a higher percentage of positive tests compared to the pre-pandemic and post-pandemic periods.
Sprague et al. [24]	United States	Late March 2020	Cohort study	Breast cancer (mammography)	Mammography screening in April 2020 was only 1.1% (0.5%, 2.4%) of what it was in April 2019. Although mammography volume rebounded in May and June, rates in June [83.8% (73.9%, 95.0%)] and July [89.7% (79.6%, 101.1%)] were still lower than pre-pandemic levels. In July, mammography volume improved the most for White [92.9% (82.9%, 104.0%) and Black women [96.7% (88.1%, 106.1%)], followed by Hispanic women [72.7% (56.5%, 93.6%)], and Asian women [51.3% (39.7%, 66.2%)]. The disparate screening rate across ethnic groups was consistent with the pre-pandemic period.
DeGroff et al. [25]	United States	Late March 2020	Cohort study	Breast (mammography) and cervical cancer (pap smears and HPV DNA)	There was an overall decrease in the volume of screening tests performed in 2020 compared to the averages of the previous five years. The sharp decline in screening began in March to April. It reached the lowest level in April 2020, with breast cancer screening declining 87% and cervical cancer screening declining 84% compared to the previous five years. The most significant decline was seen in the American Indian/Alaskan Native population for breast cancer screening (98% decline) and the Asian/Pacific Islander population for cervical cancer screening (92% decline). Although the initial decline in breast and cervical cancer was similar across rural and urban areas, urban areas saw faster improvement.
Koca and Yildirim [26]	Turkey	April 29, 2020	Cohort study	Breast cancer (mammography)	There was a 79.8% decrease in the rate of mammograms performed during the pandemic period compared to the pre-pandemic period.
Song et al. [27]	United States	Late March 2020	Cohort study	Breast cancer (mammography)	Between 2018 and 2020, the trends of mammography screening across the first ten weeks were the same. Both 2018 and 2019 also showed similar screening trends between weeks 11 and 30, with some dips during the holiday season. There was a marked decrease in mammography volume beginning week 11 of 2020, which reached the lowest level in weeks 13 to 16 when nearly zero mammograms were performed, followed by gradual improvement. At week 27, the mammography volume had recovered to about 14% below the pre-pandemic levels. On average, there was a 58% decrease in screening mammograms between weeks 11 and 30 compared to what would have been expected.
Patt et al. [28]	United States	Late March 2020	Cohort study	Breast, colon, lung, and prostate cancer	Compared to 2019, there were significant decreases in breast, colon, prostate, and lung cancer screenings in 2020. The most significant reduction was seen in breast cancer screening, which experienced an 85% decrease, followed by a 75% decrease in colon cancer screening, a 74% decrease in prostate cancer screening, and a 56% decrease in lung cancer screening.
Miller et al. [29]	United States (California)	March 19, 2020	Cohort study	Cervical cancer	There was a significant decrease in cervical cancer screening rates after the stay-at-home order was issued compared to 2019. The decrease was higher among women aged 30-65, who saw a decrease of 82% in screening rates per 100 person months, compared to women aged 21-29, who saw a decrease of 78%. In both age groups, the volume of screening tests was lowest in April 2020.
Lee et al. [30]	United States (New York)	March 20, 2020	Cohort study	Colorectal cancer (FIT)	Overall, in 2020, there was a significant decrease in the proportion of patients that had fecal immunohistochemical testing and other forms of colorectal cancer screening. In a safety-net hospital, there was a significant decrease in the proportion of outpatient visits that performed screening during the pandemic compared to the pre-pandemic period. Conversely, in a private healthcare setting, there was no significant difference in the proportion screened in 2020 compared with 2019.
Van Haren et al. [31]	United States	Late March 2020	Cohort study	Lung cancer (low-dose CT)	Low-dose CT screening was suspended during the pandemic between March 13th and May 5th, 2020, with 818 scans canceled. The 3-month and 6-month follow-up scans were prioritized upon resuming screening, while new monthly scans remained low. During the pandemic, the no-show rate for scanning significantly increased, with the most considerable no-show rate (80.6%) for annual scans, followed by 8.7% for baseline scans. No-show patients were more likely to be younger, female, African American, and current smokers.
Rutter et al. [32]	United Kingdom	March 23, 2020	Cohort study	Colorectal cancer (endoscopy)	The volume of screening flexible sigmoidoscopies began to decline at the start of March, while the volume of screening colonoscopies was maintained until mid-March. During the pandemic period, screening colonoscopies decreased by 97%, and screening sigmoidoscopies decreased by 99%.
Ferrari et al. [33]	Italy	March 9, 2020	Cohort study	Prostate cancer (PSA)	The total number of test requests for prostate cancer screening (PSA) in 2020 was the same as the previous four years; however, there was a maximal decline of 62% for PSA test requests during the lockdown period. The opposite trend (an increase in test requests) was seen for the rest of the period. After the lockdown, there was an increase of 43% in PSA test requests.
Walker et al. [34]	Canada	March 12-20, 2020	Cohort study	Multiple types of cancer	Ontario saw a decrease in screening tests of 41.3% in 2020 compared to 2019, with 72.9% fewer screenings carried out between March and May 2020.
Nyante et al. [35]	United States (North Carolina)	March 30, 2020	Cohort study	Breast cancer (mammography)	The most significant decrease in screening mammograms [-85.1% (-100.0%, -70.0%)] occurred in March 2020. The mean deficit in screening mammograms during this period was 6501 ± 1505 mammograms.
Armitage and Morling [36]	United Kingdom	March 23, 2020	Cohort study	Multiple types of cancer	During the pandemic, there was a substantial reduction in the performance metrics for four national screening programs. Breast cancer screening decreased to 55.1% during Q4 of 2020 compared to > 65% pre-pandemic. Cervical cancer screening in women younger than 50 went down to under 70% during 2020 and 2021 compared to just above 70% pre-pandemic.
Lopez de Degani et al. [37]	Argentina	March 20, 2020	Cohort study	Breast (mammography), cervical (pap smear), and colorectal (fecal occult blood)	There was a reduction in the total number of screening procedures carried out during the pandemic compared to the pre-pandemic period. The total number of pap smears went down by 56%, mammograms went down by 78.85%, and iFOBTs went down by 87.80% during the pandemic compared to the pre-pandemic period.
Ivanuš et al. [38]	Slovenia	March 16, 2020	Cohort study	Cervical cancer (cytological, histological, and HPV results)	Screening and invasive procedures decreased at the beginning of the COVID-19 pandemic, and by the fourth week, screening decreases had stabilized at around 10% below the baseline volume. HPV triage tests slowly began to improve towards the end of the pandemic period and then even went above the baseline screening volume by about 10%. The most severe deficit in screening was seen in screening smears, with the country entering the second wave of the pandemic with a screening deficit of -23%. Overall, younger women had a lower deficit of screening smears and HPV triage tests during the pandemic than older women.
Marcondes et al. [39]	United States (Massachusetts)	March 23, 2020	Cohort study	Multiple types of cancer	Pre-pandemic rates of mammography screening were lower among Asian women than White women (IR 0.82), and rates of low-dose CT were lower in Black and Latinx individuals than White women (IR 0.59 and 0.52, respectively). During the pandemic, screening rates significantly decreased across all racial and ethnic groups. By November 2020, cancer screening rates had nearly returned to pre-pandemic levels. For Latinx individuals, mammography rates in November 2020 were still lower than in 2019, while lung cancer screening was higher in 2020. Lung cancer screening rates among White and Black individuals were higher in November 2020 than pre-pandemic rates. As screening rates continued to recover, the disparities that existed pre-pandemic persisted.
Byrne et al. [42]	United States (Massachusetts and Colorado)	Late March 2020	Cross-sectional study	Lung cancer (low-dose CT)	Brigham and Women’s Hospital (Massachusetts) showed a median screening delay of 54 days, and National Jewish Health (Colorado) showed a median screening delay of 131 days during the pandemic.

The reduction in breast cancer screening across countries ranged from 9.8% to over 80% during the pandemic [12]. In 2020, the average reduction in screening uptake was 41% compared to the year before [12]. Screening disruptions in countries that experienced full lockdowns were higher than in countries that could avoid full lockdowns (relative risk of screening 0.47 v.s. 0.67, p = 0.03) [12], and studies from Europe demonstrated more significant reductions in the volume of breast cancer screening compared to those in North America [11].

On average, the United States had a 61.7% week-on-week decline in screening uptake and had the lowest levels of screening in April 2020, with estimates ranging from 1.1% [24] to 16% [25] of screening volume during the same period pre-pandemic. The United Kingdom also exhibited significant disruptions, with breast cancer screening decreasing by an average of 90% in April 2020 compared to April 2019 [16]. Turkey reported a 79.8% decrease in screening mammograms during the pandemic period compared to the pre-pandemic period [26]. Argentina similarly reported a reduction of 78.85% in screening mammograms during the pandemic period [37]. Conversely, Italy only reported a 15% reduction in mammography participation [22].

Cervical cancer screening reduced significantly across all countries beginning in March 2020, with a decrease of about 38.9% compared to the pre-pandemic periods [11]. Within the United Kingdom, between November 1, 2019, and October 31, 2020, Scotland only carried out about 43% of the tests compared to the previous year [10], while Wales’ temporary cessation of screening services involved delayed screening for 148,000 women across the region [13]. Similar to its mammogram disruption, Italy had a 15% reduction in cervical cancer screening [22], while Argentina reported a 56% reduction in pap smears during the pandemic [37]. Slovenia also experienced a significant decrease in HPV and pap smear tests, entering the second wave of the pandemic with a screening deficit of -23% compared to the previous years [38]. The United States experienced one of the larger decreases in cervical cancer screening rates. After the issuance of a stay-at-home order in California, cervical cancer screening fell by 78-82%, while other areas of the country reported an 84% decrease in April 2020 [25].

Screening services for other types of cancers were also impacted due to COVID-19 lockdowns. Colorectal cancer screening went down by 90% between January and October 2020 [11]. In Italy, colorectal screening was down by around 20% throughout 2020 [22]. In the United States, however, colorectal screening was impacted more severely, with participation reduced by 79.3% in April 2020 compared to April of the previous year [14]. The United Kingdom began to see a drop in flexible sigmoidoscopies starting in early March and a drop in colonoscopies beginning in mid-March 2020 [32]. Between 16 March to 31 May 2020, screening sigmoidoscopies and colonoscopies went down by 97% and 99%, respectively, compared to the pre-pandemic period (January 6 to March 15, 2020) [32]. Less invasive fecal occult blood tests in Argentina similarly went down by 87.8% between March 19 to September 19, 2020, compared to the same period over the previous year [37]. Other low- and middle-income countries such as Paraguay, Thailand, and Iran continued to offer different types of colorectal screening tests but only operated them at 20-90% of the expected pre-pandemic volume [14].

Across multiple countries, lung cancer screenings went down during the pandemic by about 56% [9]. Individual studies focusing on lung cancer screenings were exclusively done in the United States and identified a 75% decrease in lung cancer screening in 2020 compared to 2019 [28]. Prostate cancer screenings across countries fell by about 74% [9]. In the United States, prostate cancer screenings throughout 2020 fell by 56% compared to 2019 [28]. Italy, by contrast, maintained prostate screening volume throughout 2020 [33]. However, during the lockdown, prostate screening test requests for total prostate-specific antigen (PSA) declined by 62%, with a subsequent increase of 43% in total PSA tests above baseline after the lockdown to catch up with the deficit [33].

Disparities in Screening Disruption

Ten studies examined racial, sex, age, or socioeconomic disparities in screening disruption during the COVID-19 pandemic. While certain studies did not show any significant disparities in individuals affected by screening disruptions [35], other studies demonstrated that racial and ethnic disparities were seen in the United States and the United Kingdom [16]. The United States pre-pandemic already exhibited screening disparities, with Asian women receiving fewer mammograms than White women (incidence rate of 0.82) and Black and Latinx individuals receiving fewer low-dose CTs screening than White individuals (incidence rate of 0.59 and 0.52, respectively) [39]. These disparities were consistent following the recovery of screening services after the height of the pandemic [24]. The most significant decline in screening was seen in the American Indian/Alaskan Native population for breast cancer screening (98% decline) and the Asian/Pacific Islander population for cervical cancer screening (92% decline) [25]. By July 2020, mammography volumes had improved the most for Black and White women (92.9% of baseline), followed by Hispanic women (72.7% of baseline), and finally, Asian women (51.3% of baseline) [19]. In the United Kingdom, Black, Asian, and other minority women were more than twice as likely to delay cervical cancer screening post-pandemic due to safety fears [10].

Age and sexual preference disparities were also seen among individuals who were impacted by screening disruptions. In California, women aged between 30 and 65 had 82% lower rates of cervical cancer screening during the stay-at-home order compared to 78% lower rates in women aged between 21 and 29 [29]. A study from Slovenia demonstrated similarly that older women between the ages of 40 and 64 had more significantly disrupted cervical cancer screening compared to women aged between 20 and 39 [38]. In the United Kingdom, individuals who identified as being gay or lesbian participated less in cancer screening during the national lockdown from July to August 2020 when compared to individuals who identified as heterosexual [43].

Screen-Detected Tumors

Three studies focused on comparing the number of screen-detected tumors during the pandemic to the number detected before the pandemic period in different ways. The United States looked at the percentage of positive cases among those screened for lung nodules before and during the pandemic and noted that a higher proportion of patients screened during the pandemic had lung nodules suspicious for malignancy than before the pandemic (29% vs. 8%) [31]. Conversely, in the Netherlands, they looked at the number of screen-detected tumors compared to non-screen-detected tumors during and before the pandemic [40]. Compared to the pre-pandemic period, cancers diagnosed in 2020 were more likely to be non-screen-detected [40]. During weeks 9 to 35 of 2020, 67% fewer screen-detected tumors were diagnosed compared to the same period in previous years [40], and among 50-74-year-olds, screen-detected tumors reached a nadir of almost zero in week 14 [41].

Provision-Related Barriers

Three studies looked at provision-related barriers during the pandemic. Across multiple countries, 97% of screening centers reported disruptions due to the pandemic, and 90.9% had suspended cancer screening services [44]. Most centers stopped services in March 2020, and in most cases, the decision to suspend services was made by the government [44].

On the staffing side, 54.3% of labs surveyed experienced a shortage due to staff being deployed to support COVID-19-related care, limits set on how many staff could work on a given shift, or staff being in self-isolation [45]. About 32.6% of labs experienced delays in equipment orders, and 18.39% reported time to HPV results was significantly prolonged during the pandemic, with about two-thirds of those affected reporting delays longer than one month [45]. In the United States, over 60% of National Breast and Cervical Cancer Early Detection Program (NBCCEDP) staff were diverted to assist in the COVID-19 relief effort, and many NBCCEDP centers struggled to maintain services throughout the pandemic [19].

Patient Attitudes

Four studies looked at patient attitudes toward screening disruption during the pandemic. In the United Kingdom, 30% of women stated COVID-19 fears as their reason for being overdue for cervical cancer screening [13]. Information about safety measures in place would ease the fear in 43% of survey respondents and make them more likely to have cervical cancer screening during the pandemic [10]. Another concern for women was uncertainty about the operation of cancer screening centers during the pandemic. Some patients expected screening backlogs, and others were concerned that staff shortages would lead to male providers performing cervical cancer screening procedures [10]. By May 2020, 92% of respondents wanted the government to reinstate screening programs [13], and over 60% felt the pandemic had impacted their cancer care [46].

In the United States, over 90% of patients thought it was appropriate to delay lung cancer screening due to the increasing number of COVID-19 cases [42]. Most felt it would not negatively impact their future screening progress or mental health [42]. Opinions on being exposed to COVID-19 were somewhat mixed, with 41% strongly disagreeing that attending screening would increase their risk of contracting the virus and 29% feeling that it would increase their risk [42].

Discussion

The primary objective of this review was to assess the impact of COVID-19 lockdowns on worldwide cancer screening uptakes. Almost all 31 studies examining this outcome demonstrated significant decreases in cancer screenings across multiple countries. Countries hit more severely by COVID-19 had higher rates of disruption in cancer screenings [9]. As expected, countries with lockdowns also experienced more significant disturbances to cancer screenings than those that did not experience lockdowns [12].

The secondary objectives of this review were to determine whether there were disparities in screening disruption, changes in the number of screen-detected tumors, provider barriers to screening, and patient attitudes toward screening. Racial disparities were mainly seen in the United States and the United Kingdom. In both countries, racial and ethnic minorities had longer delays or slower resumption of screening than White individuals. Interestingly, two separate studies in the United States showed Asian women being one of the most significantly impacted minority groups during and after the height of the pandemic [25,39]. The reasons for this are not explored but may be related to socioeconomic or cultural factors. On the provider side, there were multiple barriers, including staff-related shortages and factors influencing the availability of equipment, which may be due to significant supply-side shortages and global disruptions in business due to the pandemic. Patients seemed to understand the initial pauses or delays in screenings, and many opted to delay screenings due to fear of contracting the virus. However, as time passed and the fear of contracting the virus decreased, patients began to worry about the negative consequences of missed or delayed screenings. By then, patients instead hoped screening services could be resumed with appropriate sanitization and distancing measures.

Limitations

This study focused primarily on countries that underwent a complete lockdown; however, despite not going through a total lockdown, many other countries still had cancer screening services impacted by the COVID-19 pandemic. These impacts were not assessed in the scope of this study. Furthermore, some countries, such as China, faced continuous lockdowns until very recently and may continue to face disruptions to their services which have yet to be thoroughly studied. Included studies are not equally distributed in terms of the cancer type and country. Although several included reviews look at multiple countries of various income levels, only one paper looked specifically at a low or middle-income country, Bangladesh, compared to thirteen studies that assessed impacts in the United States alone. There was also an unequal distribution of the cancer types screened, with breast cancer being looked at in ten studies compared with just one study on prostate cancer. This unequal distribution may be due to different national screening programs across various countries, with breast and cervical cancer being the most widely screened.

Future Research

Future research on this topic is essential to continue to update our evolving knowledge base on the impact of pandemic lockdowns on cancer screening disruptions. Further research is needed from low- and middle-income countries to complement the data from high-income countries and give a more balanced view of the issue. When appropriate, further research on screening for cancers other than breast and cervical cancer can also provide a more comprehensive picture of the problem. Other aspects that need further research are the barriers certain minority groups face that lead to much lower screening rates before, during, and after the pandemic and the psychological and physical impacts screening disruptions have had on patients.

Possible Policy Implementations

The studies reviewed also explored various ways to implement policies and guidelines to deal with the inevitable disruptions to cancer screenings that may arise if we ever have another lockdown. One possible way is to incorporate more self-sampling into the guidelines. Some studies showed that during COVID-19, self-sampling was a way to maintain screening volume [45]. In addition, studies from low- and middle-income countries, such as Nepal, have also demonstrated successful trials with self-sampling [47]. There are limitations with self-sampling as it can only be used in certain types of cancers, such as colorectal and cervical cancer, and self-sampling may not translate to higher follow-up rates. Other policies can target risk stratification so that even if services are reduced, high-risk women are still offered timely screening while screening intervals for lower-risk individuals are extended. Finally, as artificial intelligence and digitalization become more prominent in healthcare, we may develop new tools for better distancing and at-home methods to improve uptake without increasing patients’ risk of infection.

## Conclusions

This review aimed to synthesize evidence on the evolving field of the indirect health consequences of the pandemic, specifically in terms of cancer screening. These findings, while non-exhaustive, demonstrate the significant impacts the pandemic has had on screening uptake and screen-detected tumors while shedding light on demographic discrepancies, provider-related barriers, and patient attitudes and concerns regarding these disruptions. Providing a more comprehensive global picture will require additional information about less widely investigated cancers and data from less researched parts of the world. Understanding the extent and mechanisms of disruption on a global scale will help us identify specific pain points to target as we improve our screening delivery in the aftermath of COVID-19 and plan for future crises.
